# The Prenatal Diagnosis and Clinical Outcomes of Fetuses With 15q11.2 Copy Number Variants: A Case Series of 36 Patients

**DOI:** 10.3389/fmed.2021.754521

**Published:** 2021-11-23

**Authors:** Jessica Kang, Chien-Nan Lee, Yi-Ning Su, Ming-Wei Lin, Yi-Yun Tai, Wen-Wei Hsu, Kuan-Ying Huang, Chi-Ling Chen, Chien-Hui Hung, Shin-Yu Lin

**Affiliations:** ^1^Department of Obstetrics and Gynecology, National Taiwan University Hospital, Taipei, Taiwan; ^2^Department of Medical Genetics, National Taiwan University Hospital, Taipei, Taiwan; ^3^Sofiva Genomics Co. Ltd., Taipei, Taiwan; ^4^Department of Obstetrics and Gynecology, National Taiwan University Hospital Hsin-Chu Branch, Hsinchu, Taiwan; ^5^Department of Obstetrics and Gynecology, National Taiwan University Hospital Hospital Yun-Lin Branch, Yunlin, Taiwan

**Keywords:** 15q11.2 microdeletion, 15q11.2 microduplication, BP1–BP2, copy number variant, chromosome microarray analysis (CMA), prenatal, neurodevelopment

## Abstract

Prenatal genetic counseling of fetuses diagnosed with 15q11.2 copy number variants (CNVs) involving the BP1–BP2 region is difficult due to limited information and controversial opinion on prognosis. In total, we collected the data of 36 pregnant women who underwent prenatal microarray analysis from 2010 to 2017 and were assessed at National Taiwan University Hospital. Comparison of the maternal characteristics, prenatal ultrasound findings, and postnatal outcomes among the different cases involving the 15q11.2 BP1–BP2 region were presented. Out of the 36 fetuses diagnosed with CNVs involving the BP1–BP2 region, five were diagnosed with microduplications and 31 with microdeletions. Among the participants, 10 pregnant women received termination of pregnancy and 26 gave birth to healthy individuals (27 babies in total). The prognoses of 15q11.2 CNVs were controversial and recent studies have revealed its low pathogenicity. In our study, the prenatal abnormal ultrasound findings were recorded in 12 participants and were associated with 15q11.2 deletions. No obvious developmental delay or neurological disorders were detected in early childhood.

## Introduction

Copy number variants (CNVs) involving chromosome 15q11-q13 is a challenging issue for prenatal counseling. Prader–Willi syndrome (PWS), Angelman syndrome (AS), and 15q11-q13 duplication syndrome were known as the three most studied neurodevelopmental disorders occurring at the locus (1). The 15q11.2 CNV involving non-imprinting breakpoints 1–2 (BP1–BP2) is included in the category of incidental finding not to be reported due to no definite linkage to phenotype and low penetrance (2), and a recent study has also concluded with low pathogenicity of this region (3). However, several cases presented with a wide variety of neuropsychiatric disorders have been reported, and this region was strongly associated with neurodevelopmental disorders while reviewing previous literature (4–6). In Asia, few studies have been conducted specifically in the region involving BP1–BP2 (7, 8), while a case reported with recurrent microdeletion encompassing BP1–BP2 region was also presented with developmental and motor delay (9).

There are five common breakpoints within 15q11-q13, defined as BP1 through 5. The most common breakpoints involved with the deletions are BP1, BP2, and BP3 (1). The classic PWS/AS deletion is flanked by either the proximal BP1 or BP2 breakpoints and the distal BP3 breakpoint, which generally leads to severe symptoms. The cases diagnosed with 15q11.2 BP1–BP2 deletions alone are usually presented with less severe symptoms or even non-symptomatic (10). However, it is interesting that the PWS/AS cases with type I deletion were presented with more severe neurodevelopmental disorders than the cases with type II deletion. This finding has indicated that BP1–BP2 region might affect the phenotypes in the patients with PWS/AS (11). Various clinical manifestations have been detected in the cases with 15q11.2 CNVs, such as motor delay, intrauterine growth retardation, macrocephaly, non-specific dysmorphic features (1, 10), congenital cataracts, esophageal atresia (12), and arthrogryposis (13).

The CNV involving the BP1–BP2 region is more challenging in prenatal counseling due to its incomplete penetrance with variable expressivity. The four genes (TUBGCP5, CYFIP1, NIPA1, and NIPA2) within the BP1–BP2 region have been noted to affect the clinical presentation and severity of neurological impairment, and this region is ~500 kb in size (7). The tubulin gamma complex associated protein 5 (TUBGCP5) gene is related to neurobehavioral disorders, such as attention deficit hyperactivity disorder and obsessive-compulsive disorder (14). Cytoplasmic fragile X mental retardation 1 interacting protein 1 (CYFIP1) gene is a member of the Wave regulatory complex and regulates actin remodeling during neural wiring. CYFIP1 gene product also interacts with Fragile X mental retardation protein (FMRP) in a ribonucleoprotein complex, which regulates the translation of FMRP-target messenger RNAs and has been noted to be responsible for Fragile X syndrome (15). Both non-imprinted in Prader-Willi/Angelman syndrome 1 (NIPA1) gene and non-imprinted in Prader-Willi/Angelman syndrome 2 (NIPA2) gene regulate magnesium transport. NIPA1 is associated with autosomal dominant hereditary spastic paraplegia (16–18), while NIPA2 is reported to be associated with childhood absence epilepsy (19, 20).

The overall prevalence of 15q11.2 CNVs involving BP1-BP2 was reported to be 0.5–1% (21, 22). Although the deletions and duplications of the BP1–BP2 region are equally common, the previous studies have reported that the deletions have a more severe impact than duplications (10), with clinical features presenting as cognitive deficits, motor delays, autism spectrum disorder (ASD), ataxia, attention disorders, and seizures (7, 23). Other psychiatric problems, such as schizophrenia, obsessive-compulsive disorder, and oppositional defiant disorder (24). The effect of BP1–BP2 region on cognitive function is reported to be more pronounced in the deletion carriers since cognitive impairment was noted in the unaffected carriers of the 15q11.2 deletion and not in duplication carriers (25, 26). Individuals diagnosed with 15q11.2 BP1-BP2 duplication are reported having developmental delay, motor or language delay, epilepsy, learning disabilities, and behavioral issues (4, 5, 10). However, in the recent large-scale genetic studies, the duplication was not defined as a risk locus for schizophrenia and developmental delay, and its role in autism should also be interpreted with caution.

Variable penetrance of this CNV is reported (8, 27). According to the previous studies, the *de novo* frequency of 15q11.2 BP1–BP2 microdeletion is around 5−22%. About 80% of the cases are reported to be inherited from their parent (7), such as 50% inherited from an apparently unaffected parent and 35% inherited from an affected parent (1). Different origins of inheritance are associated with different phenotypes (24). As for duplication, no previous statistics on the inheritance pattern have been collected, and the information about their prognosis was extremely limited (5, 28, 29).

The uncertainty of both penetrance and the severity of phenotypes have increased the complexity of prenatal genetic counseling, especially in fetuses diagnosed with abnormal prenatal ultrasound. Therefore, we retrospectively reviewed 36 cases that were diagnosed with 15q11.2 CNVs involving the BP1–BP2 region, aiming to find possible correlation between phenotype and abnormal sonographic findings, and compare with previously reported cases.

## Materials and Methods

The prenatal microarray analyses of total of 15,051 cases were assessed at National Taiwan University from July 1, 2012 to December 31, 2017. We collected data from 36 pregnant women, whose microarray analyses showed that the fetuses were carrying CNVs involving the 15q11.2 BP1–BP2 region. Clinical information and pregnancy outcomes were collected from the medical records, such as maternal characteristics, family history, indications for invasive testing, prenatal ultrasound finding, delivery mode, newborn characteristics, and developmental follow-up. Indications for invasive testing include advanced maternal age, karyotype abnormalities, abnormal ultrasound findings, and maternal anxiety. Microarray data of all cases were analyzed retrospectively for microdeletion and microduplication involving the 15q11.2 BP1-BP2 region. Initial developmental status of the cases delivered was assessed by the pediatricians during regular follow-up for vaccination.

The participants underwent amniocentesis or chorionic villus sampling, where 10 ml of amniotic fluid or chorionic villi was sampled through abdominal puncture under ultrasound guidance. Once received, genomic DNA was extracted from the amniotic fluid or chorionic villi using the DNA Extraction Kit (QIAamp® DNA Blood Mini Kit, QIAGEN, Hilden, Germany), according to the instructions of the manufacturer.

All the research methods used in this process were approved by the National Taiwan University Hospital Research Ethics Committee (201801010RINC), Taipei, Taiwan.

### Cytogenomic Microarray Analysis

The 8 × 60 K oligonucleotide array (Agilent Technologies, Santa Clara, CA, USA) and the Affymetrix CytoScan 750 K single nucleotide polymorphism (SNP) array analysis (Affymetrix Inc., Santa Clara, CA, USA) were used, and all the procedures were carried out according to the protocols of the manufacturer.

#### Array Comparative Genomic Hybridization (CGH) Analysis

The SurePrint G3 Human CGH Microarray Kit 8 × 60 K (Agilent Technologies, Santa Clara, CA, USA) was used. DNA extraction was performed using the QIAamp DNA Blood Mini Kit (QIAGEN, Hilden, Germany). The slides were scanned using the SureScan Microarray Scanner (Agilent Technology, Santa Clara, CA, USA) and analyzed with Feature Extraction Software v11.5 (Agilent Technology, Santa Clara, CA, USA) under designed parameters of the human reference genome hg19. The data analysis was conducted *via* the Agilent Cytogenomics software available on the website of the company (https://www.genomics.agilent.com/en/CGH-Microarray-Data-Analysis/CytoGenomics-Software/?cid=AG-PT-111&tabId=AG-PR-1017, Agilent Cytogenomics v2.7.8.0).

#### SNP Array Analysis

The Affymetrix CytoScan 750 K SNP array analysis (Affymetrix Inc., Santa Clara, CA, USA) was employed, with a size threshold of 400 kb used for all CNVs. All the procedures were carried out according to the protocols of the manufacturer. The sample DNA (250 ng) was digested, ligated, and amplified by using PCR, followed by purification, fragmentation, labeling, hybridization, dyeing, and scanning. The data analysis was performed using Chromosome Analysis Suite (ChAS) software (v3.1, r8004).

### Statistical Analysis

The Mann–Whitney *U*-test was applied to calculate the maternal age difference between deletion and duplication groups. The Chi-squared test was applied to test the relationship between different fetal gender, parental origin of inheritance, and type of CNVs (deletion and duplication) with the presence of ultrasound abnormality, and used to compare the relationship between 15q11.2 deletion and abnormal prenatal ultrasound findings. The significance level was set at *p* < 0.05 (two-sided). The statistical analyses were performed using SPSS V22.0 software.

## Results

Although different microarray platforms were used in our study, the SNP microarray analysis was used with the majority of our subjects. Of all 36 cases, we screened nine cases using the 60 K oligonucleotide array (Agilent Technologies Inc., Santa Clara, CA, USA), and 27 cases with the Affymetrix CytoScan 750 K SNP array analysis (Affymetrix Inc., Santa Clara, CA, USA).

Of all 15,051 cases, 1,577 cases received microarray analysis due to abnormal prenatal ultrasound findings, and the indication of the remaining 13,474 cases was advanced maternal age, prior family history, and maternal anxiety. Among the 36 cases detected with 15q11.2 CNVs, five cases were diagnosed with microduplication and 31 cases with microdeletion in this cohort, which represent 0.03% (5/15,051) and 0.21% (31/15,051) of the cases analyzed, respectively. There was no difference in maternal age between the deletion and duplication groups (*p* = 0.4). In addition, we compared the relationship among the fetal gender, parental origin of inheritance, type of CNVs (deletion and duplication) with the presence of ultrasound abnormality. The results showed no significant difference in fetal sex (*p* = 0.548), parental origin (*p* = 0.33), and the type of CNV (*p* = 0.414).

Ten participants underwent termination of pregnancy, while 26 participants (25 deletion carriers and 1 duplication carrier) delivered 27 healthy babies (one case was multipara and delivered two babies as deletion carriers in this period) without further complications.

### 15q11.2 Microduplication

**Figure 1:**
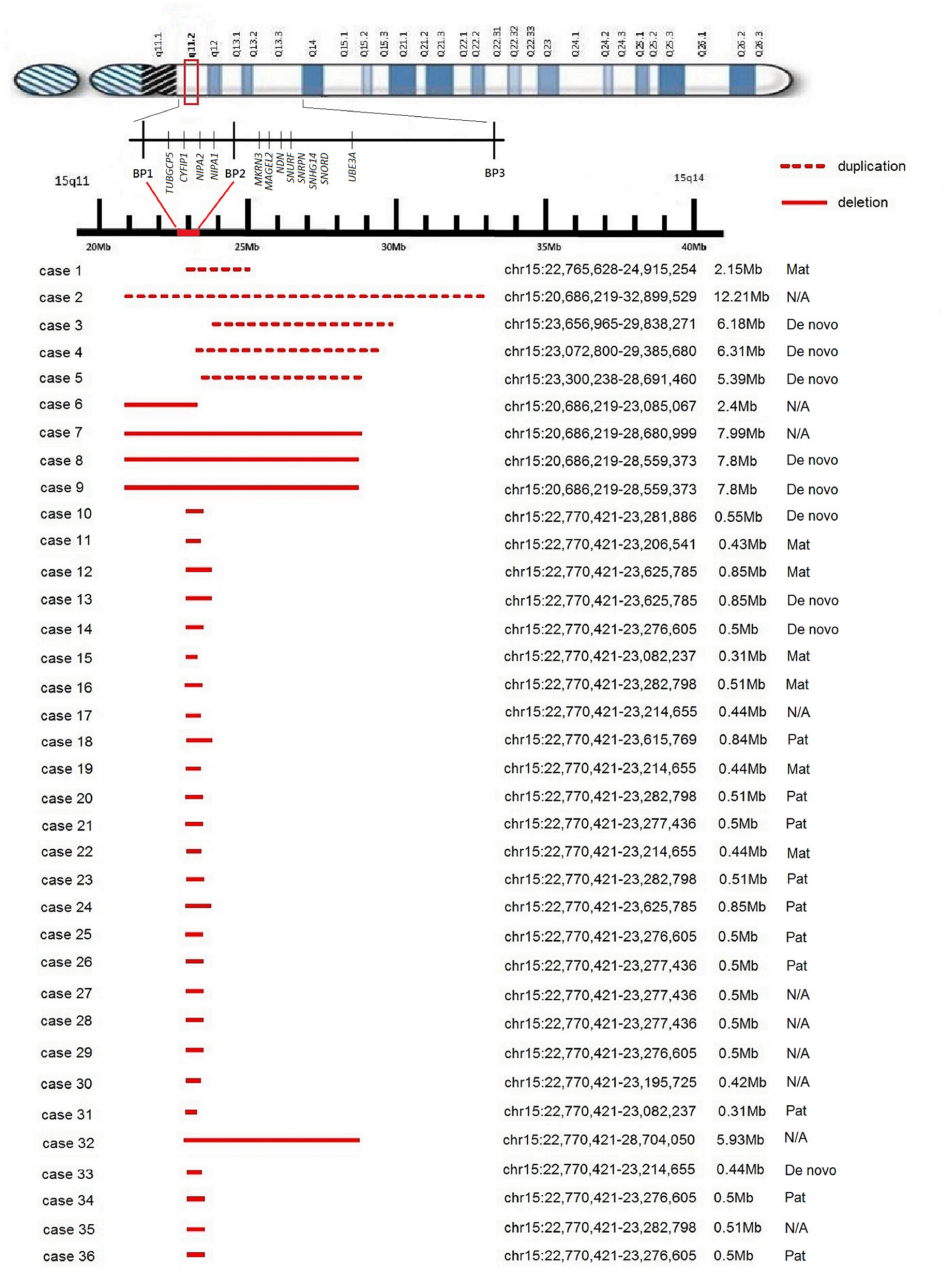
Schematic map of the 15q11.2 BP1-BP2 region. The reported microduplications and microdeletions are shown at the bottom drawn to scale.

We identified a duplication within the 15q11.2 BP1-BP2 region in five cases. Only one case involved the four highly conserved genes (Figure 1), and the size of duplication ranged from 2.15 to 12.21 Mb of chromosome 15. Three cases were proven to be *de novo*, while the other one was maternal in origin. Case 2 was of unknown origin because further study was not conducted.

One case was delivered at term without major anomalies or complications. Four cases underwent termination of pregnancy due to the involvement of the PWS/AS region (case 2–5), with one diagnosed with tetralogy of Fallot prenatally.

For cytogenetic findings and details (see Figure 1). The clinical details are listed in Table 1.

**Table 1:** The findings of fetuses with 15q11.2 copy number variant (CNV) and newborn characteristics.

**Case**	**Sex**	**Dup/del**	**Size (Mb)**	**Origin**	**Prenatal ultrasound finding**	**Growth IUGR**	**Delivery mode**	**Gestational age at birth**	**Birth body weight (g)**	**Apgar score**	**Postnatal finding**	**Follow-up years**	**DD**
1	M	Dup	2.15 Mb	Maternal		-	C/S	38+2	2,994	9–9		3	-
2	F	Dup	12.21 Mb	N/A		-	Termination	22	445				N/A
3	M	Dup	6.18 Mb	*De novo*		-	Termination	21	405				N/A
4	F	Dup	6.31 Mb	*De novo*		-	Termination	27+6	730				N/A
5	M	Dup	5.39 Mb	*De novo*	Tetralogy of Fallot	-	Termination	23+4	495				N/A
6	M	Del	2.4 Mb	N/A		-	VD	38+4	2,840	9–9		8	-
7	M	Del	7.99 Mb	N/A		-	Termination	23	480				N/A
8	F	Del	7.8 Mb	*De novo*	Chylothorax with fetal hydrops	-	Termination	23+4	850		Hydrops fetalis		N/A
9	F	Del	7.8 Mb	*De novo*	Total anomalous pulmonary venous return	-	Termination	26	580				N/A
10	F	Del	0.55 Mb	*De novo*	Ventricular septal defect	-	VD	38+4	3,125	8–9		4	-
11	M	Del	0.43 Mb	Maternal	Echogenic intracardiac focus	-	C/S	38+2	2,620	8–9		4	-
12	F	Del	0.85 Mb	Maternal	Left duplicated kidney	-	C/S	38+1	3,080	8–9		3	-
13	M	Del	0.85 Mb	*De novo*	Ventricular septal defect	-	C/S	32+1	1,840	7–8		2	-
14	F	Del	0.5 Mb	*De novo*		-	VD	39+1	2,645	8–9		5	-
15	F	Del	0.31 Mb	Maternal		-	VD	40+1	3,780	8-−9		3	-
16	F	Del	0.51 Mb	Maternal		-	C/S	31+3	1,740	6–8		2	-
17	F	Del	0.44 Mb	N/A		-	VD	39+5	3,310	9–10		4	-
18	M	Del	0.84 Mb	Paternal		-	VD	39+2	2,276	8–9		2	-
19	F	Del	0.44 Mb	Maternal	Hypoplastic left heart syndrome	-	Termination	22+6	540				N/A
20	M	Del	0.51 Mb	Paternal	Single umbilical artery	-	VD	39+1	3,040	9–9		2	-
21	F	Del	0.5 Mb	Paternal		-	VD	39+4	2,844	9–10		2	-
22	M	Del	0.44 Mb	Maternal	Fetal ascites, echogenic bowel	-	C/S	38+1	3,320	9–10		2	-
23	M	Del	0.51 Mb	Paternal		-	VD	27+2	884	6–8		2	-
24	F	Del	0.85 Mb	Paternal	Ventricular septal defect	-	VD	39+5	2,986	9–9		2	-
25	M	Del	0.5 Mb	Paternal		-	VD	40	3,522	9–9		2	-
26	F	Del	0.5 Mb	Paternal		-	VD	35+4	2,296	8–9		2	-
27	M	Del	0.5 Mb	N/A		-	C/S	39+2	3,110	9–9		4	-
28	M	Del	0.5 Mb	N/A		-	VD	39+3	3,630	9–10		3	-
29	M	Del	0.5 Mb	N/A		-	C/S	37+6	2,734	9–9		4	-
	F	Del	0.5 Mb	N/A		-	C/S	37+4	4,070	9–10		2	-
30	F	Del	0.42 Mb	N/A		-	VD	37	2,534	9–10		3	-
31	F	Del	0.31 Mb	Paternal		-	VD	38+1	2,884	9–10		4	-
32	M	Del	5.93 Mb	N/A		-	Termination	21+2	360				N/A
33	F	Del	0.44 Mb	*De novo*		-	VD	39	3,210	9–10		5	-
34	F	Del	0.5 Mb	Paternal	Oligohydramnios	-	C/S	40	3,075	9–10		3	-
35	M	Del	0.51 Mb	N/A	Nuchal thickness 5.2 mm	-	Termination	13+5	46		Nuchal edema		N/A
36	M	Del	0.5 Mb	Paternal		-	VD	40+1	3,310	8–9		2	-

*Dup, duplication; Del, deletion; N/A, not applicable; VD, vaginal delivery; C/S, cesarean section; IUGR, intrauterine growth restriction; DD, developmental delay*.

### 15q11.2 Microdeletion

We identified microdeletion of 15q11.2 BP1-BP2 region in 31 cases. The deletion involved the four highly conserved genes in 30 cases (Figure 1), and only one involved partially, ranging from 0.31 to 7.99 Mb of chromosome 15. Five of the microdeletion cases were proven to be *de novo*, six were maternal and nine were paternal in origin, while 11 were of unknown origin.

Among all 31 cases diagnosed with 15q11.2 microdeletion, 12 cases (prevalence 0.79%, 12/15,051) were diagnosed with abnormal ultrasound prenatally (such as fetal malformation, increased nuchal translucency, soft markers, and oligohydramnios, details are listed in Table 2), and 19 (prevalence 0.13%, 19/15,051) were diagnosed without abnormal prenatal ultrasound. We compared the four different groups: (1) 15q11.2 deletion with abnormal ultrasound finding (*N* = 12); (1) 15q11.2 deletion with normal ultrasound finding (*N* = 19); (3) normal array result with abnormal ultrasound finding (*N* = 1,189); and (4) normal array result with normal ultrasound finding (*N* = 13,199) and calculate the relationship by chi-squared test *via* SPSS. The result showed that the presence of 15q11.2 microdeletions is related to abnormal prenatal ultrasound (*p* < 0.05).

**Table 2:** Different types of abnormal ultrasound were detected in 15q11.2 microdeletion cases.

**Type**	**Cases with deletion *N* (%)**
**Malformation**	
Cardiovascular	5 (16.1%)
Genitourinary	1 (3.2%)
Soft markers	3 (9.7%)
Increased nuchal translucency (NT>3 mm)	1 (3.2%)
Oligohydramnios	1 (3.2%)
Others	1 (3.2%)

Six cases underwent termination of pregnancy, and abnormal ultrasound findings were reported in four cases prenatally including one with fetal chylothorax, two with congenital cardiac disease, and one with nuchal edema.

The other 25 participants continued their pregnancy, with 4 delivering preterm due to obstetric complications (ranging from 27 to 35 weeks of gestational age) and 21 cases (22 deliveries) delivered at term without complications. Of the 25 cases delivered, the abnormal ultrasound findings were confirmed in seven cases prenatally, such as three cases of ventricular septal defect, one of duplex kidney, one of single umbilical artery, one of fetal ascites, and one of oligohydramnios.

## Discussion

In this study, total of 36 cases were diagnosed with 15q11.2 CNVs involving the BP1–BP2 region. The presence of prenatal abnormal ultrasound finding was considered related to 15q11.2 deletions. Most of the cases choose to deliver their baby and there was no obvious developmental delay or neurological disorders detected in early childhood.

Prenatal genetic diagnosis has become a trend due to advanced maternal age, while the progress in genetic testing resolution provides more detailed information to clinicians. A microarray analysis is effective in screening for submicroscopic genomic imbalance and may expand the scope of diagnosis by 8.2% compared with conventional karyotyping for those with abnormal ultrasound results (30). The clinical interpretations of the rare cases of microdeletion, microduplication, and variants of unknown significance (VOUS) have also been a challenge. CNVs of 15q11.2 have always been a difficult issue for prenatal genetic counseling due to incomplete penetrance and variant phenotype expression. This CNV is previously considered a risk locus and there have been some reviews investigating 15q11.2 microdeletion worldwide, but general population-based data are still lacking (8, 10, 14). As for microduplication, even less information can be found as it has not been extensively studied. Recently, a report in Israel that includes 160 cases diagnosed with 15q11.2 CNVs either prenatally or postnatally suggested this region has low pathogenicity (3).

In Taiwan, there has been a recent study reviewing adverse perinatal and early life outcomes in the 15q11.2 CNV carriers. The deletion carriers are reported to be more symptomatic and have higher neonatal intensive care unit (ICU) transfer, while our study has not shown such tendency. Interestingly, Chu et al. have mentioned that the prevalence of congenital heart disease was also higher in the deletion group, and most of our ultrasound abnormalities in the deletion group are cardiovascular (31). The other two case reports were cases with 15q11.2 BP1–BP2 duplication. One case has been diagnosed with ventriculomegaly, microcephaly, and intrauterine growth restriction and underwent termination (32). Another case who delivered, in the end, had undergone amniocentesis for fetal karyotyping, which revealed 46, XX. However, developmental delay was noted in this baby and two siblings. Further genetic study revealed the 15q11.2 duplication was inherited from their phenotypically normal father. Thus, incomplete penetrance is also a challenge regarding 15q11.2 duplication, as a wide variety of phenotypes may be presented in the same family (9). In our study, only one case of duplication (case 1) was inherited from a phenotypically normal mother. The 2.15 Mb duplication of this case is involved with makorin ring finger protein 3 (MKRN3) gene presented with precocious puberty and Schaff–Yang syndrome related to melanoma antigen family L2 (MAGEL2) gene mutation. No developmental delay was noted in the following early childhood. Four out of five duplication cases received termination due to large duplication size with involvement of the PWS/AS region, thus no detailed information on the penetrance and expressivity was available, and the clinical significance of 15q11.2 duplication is still uncertain.

Many healthy individuals were reported with 15q11.2 BP1–BP2 deletion incidentally. However, this region is associated with developmental delay and behavioral disorders in phenotypically abnormal cases (7, 10, 33). The estimated risk of an abnormal phenotype in 15q11.2 deletions ranges from 10.4 to 83% (34, 35). According to Kirov et al., the frequency of 15q11.2 deletions in the general population is 0.3%, while the penetrance for schizophrenia is around 2%, and the penetrance for ASD, DD, and various congenital malformations is around 11% (36). There have been few data reported in the prenatal population, while our study focused on this particular group that underwent invasive genetic testing. The prevalence of 15q11.2 CNVs and the disease penetrance in the originally non-phenotypic group are the main focus. If the 15q11.2 CNVs seems to be a risky or pathogenic locus, the parents might have different thoughts of whether they would continue the pregnancy or not.

Among the cases of known inheritance patterns, the percentage of *de novo* mutation, maternal origin, and paternal origin were 35, 27, and 38%, respectively. The majority of the deletion cases chose to deliver their fetuses. Case 6 with *de novo* microdeletion of 15q11.1-q11.2 delivered a healthy baby without further early childhood developmental disorders. The array report of case 6 showed a relatively small deletion size (2.4 Mb) although involved with the 15q11.2 BP1-BP2 region and four genes. The other 25 participants who delivered healthy individuals had a relatively small deletion size (ranging from 0.31 to 0.85 Mb). Most of the cases that underwent termination in the microdeletion group were found to be having large deletion size and involved with the PWS/AS region. Four cases of congenital anomalies were diagnosed *via* prenatal ultrasound scanning, such as one diagnosed with Down syndrome by chorionic villus sampling. Although most cases of microdeletion were delivered without serious complications, the time for follow-up is relatively short. Long-term growth development and evaluation should be conducted in the future.

Another interesting issue is whether there is a relationship between the specific ultrasound features and 15q11.2 CNVs. Not all the pregnant women would receive amniocentesis for genetic study at the beginning. The abnormal ultrasound findings were reported in some cases and needed further evaluation. Some specific features might be related to specific chromosomal abnormalities or genetic syndromes. Dysmorphic feature (43%) is the most common sonographic characteristic noted in the cases of chromosomal abnormalities, which was also noted in the previous studies of 15q11.2 deletion, and cardiac diseases were also found in 10–20% of the cases of 15q11.2 deletion (27). The cardiac problems reported include complex left-sided malformations, atrial and ventricular septal defects, coarctation of the aorta, and tetralogy of Fallot. In our study, 12 cases of microdeletion had abnormal ultrasound findings diagnosed prenatally, such as six with congenital cardiac defects, one with chylothorax associated with hydrops fetalis, one with a duplex kidney, one with isolated single umbilical artery, one with fetal ascites with echogenic bowel, one with nuchal thickening, and one with oligohydramnios. For those with heart defects, the one with total anomalous pulmonary venous return and the one with hypoplastic left heart syndrome underwent termination of pregnancy. None of the cases had dysmorphic features. In our study, we compared the presence of abnormal prenatal ultrasound and 15q11.2 deletion in different groups and found out an association, while the result is not compatible with the recent Israelis findings (3). However, the result should be interpreted with caution due to the variety of different types of prenatal ultrasound findings. There was no previous study investigating 15q11.2 duplications with the abnormal ultrasound findings, and only one out of five cases of duplication was diagnosed with tetralogy of Fallot *via* ultrasound examination in this study. The incidence of congenital heart disease is too low, while a previous study found that the detection rate in an unselected population is ~16.9% (37). In summary, an abnormal prenatal ultrasound finding is associated with 15q11.2 deletions.

The first limitation of this study is the relatively small case number of diagnosed CNVs. The prevalence of 15q11.2 deletions and duplications among the prenatal test is ~0.3–0.5% and 0.8% (3, 36). The prevalence of deletions (0.21%) and duplications (0.03%) in our study seems to be lower than the data reported in previous literature. If we could collaborate with other medical centers or hospitals academically in the future, the general population would be larger and might have more cases with abnormal prenatal genetic results. The larger study population may provide more information for us to offer a more detailed explanation to the pregnant woman. Second, the follow-up period of the offspring is too short. The disorders with intellectual or learning disabilities and behavioral issues, such as autism might be missed. Thus, we should expand the interval of follow-up, so that the growth development could be evaluated more thoroughly in the future.

The prognostic accuracy of 15q11.2 CNVs was mostly unknown because some cases underwent termination of pregnancy. In our study, no obvious developmental delay or neurological disorders in early childhood were detected in the one case of 15q11.2 microduplication and 25 cases of microdeletion. Prenatal abnormal ultrasound is associated with 15q11.2 deletions involving BP1–BP2. However, the prevalence of 15q11.2 CNVs is very low in the Taiwanese population, which suggests that our findings should be interpreted with caution and indicates the need for studies that include large numbers of control subjects to ascertain the impact.

## Data Availability Statement

The raw data supporting the conclusions of this article will be made available by the authors, without undue reservation.

## Ethics Statement

The studies involving human participants were reviewed and approved by National Taiwan University Hospital Research Ethics Committee (201801010RINC). Written informed consent for participation was not required for this study in accordance with the national legislation and the institutional requirements.

## Author Contributions

JK contributed to the drafting of the main manuscript. M-WL, Y-YT, and C-LC organized and analyzed the patient data. W-WH, K-YH, and C-HH drew and formatted the tables and the figure. C-NL and Y-NS participated in the study design and data collection. S-YL conceived the study and helped revise the manuscript. All authors read and approved the final manuscript.

## Funding

This work was supported by the Ministry of Science and Technology under the grant 108-2314-B-002-143-MY3 and the National Taiwan University Hospital under the grant NTUH108-N4040.

## Conflict of Interest

Y-NS was employed by the company Sofiva genomics Co. Ltd. The remaining authors declare that the research was conducted in the absence of any commercial or financial relationships that could be construed as a potential conflict of interest.

## Publisher's Note

All claims expressed in this article are solely those of the authors and do not necessarily represent those of their affiliated organizations, or those of the publisher, the editors and the reviewers. Any product that may be evaluated in this article, or claim that may be made by its manufacturer, is not guaranteed or endorsed by the publisher.
